# Adaptation in the Visual Cortex: Influence of Membrane Trajectory and Neuronal Firing Pattern on Slow Afterpotentials

**DOI:** 10.1371/journal.pone.0111578

**Published:** 2014-11-07

**Authors:** Vanessa F. Descalzo, Roberto Gallego, Maria V. Sanchez-Vives

**Affiliations:** 1 IDIBAPS (Institut d'Investigacions Biomèdiques August Pi i Sunyer), Barcelona, Spain; 2 Instituto de Neurociencias de Alicante, Universidad Miguel Hernández-CSIC, Alicante, Spain; 3 ICREA (Institucio Catalana de Recerca i Estudis Avançats), Barcelona, Spain; University Paris 6, France

## Abstract

The input/output relationship in primary visual cortex neurons is influenced by the history of the preceding activity. To understand the impact that membrane potential trajectory and firing pattern has on the activation of slow conductances in cortical neurons we compared the afterpotentials that followed responses to different stimuli evoking similar numbers of action potentials. In particular, we compared afterpotentials following the intracellular injection of either square or sinusoidal currents lasting 20 seconds. Both stimuli were intracellular surrogates of different neuronal responses to prolonged visual stimulation. Recordings from 99 neurons in slices of visual cortex revealed that for stimuli evoking an equivalent number of spikes, sinusoidal current injection activated a slow afterhyperpolarization of significantly larger amplitude (8.5±3.3 mV) and duration (33±17 s) than that evoked by a square pulse (6.4±3.7 mV, 28±17 s; p<0.05). Spike frequency adaptation had a faster time course and was larger during plateau (square pulse) than during intermittent (sinusoidal) depolarizations. Similar results were obtained in 17 neurons intracellularly recorded from the visual cortex *in vivo*. The differences in the afterpotentials evoked with both protocols were abolished by removing calcium from the extracellular medium or by application of the L-type calcium channel blocker nifedipine, suggesting that the activation of a calcium-dependent current is at the base of this afterpotential difference. These findings suggest that not only the spikes, but the membrane potential values and firing patterns evoked by a particular stimulation protocol determine the responses to any subsequent incoming input in a time window that spans for tens of seconds to even minutes.

## Introduction

A large number of studies quantifying neuronal sensory responses have been carried out by means of extracellular recordings from anesthetized or awake chronically implanted animals. Most often the effect of sensory stimulation has been measured by the number, frequency and distribution of action potentials or by input/output relationships; that is, as the suprathreshold evoked response. This has strengthened the view that synaptic transmission, passive membrane properties and relatively fast membrane conductances are the main determinants of neuronal integration. Evidence of it is the extended use of integrate and fire network computational models centered on the suprathreshold properties of neurons [1], for a review see [2], [3]. As a result, the influence of the membrane potential trajectory underlying a sensory response, especially when of long duration, has often been neglected.

The membrane potential following visual responses can depart from the resting potential for long periods of time spanning tens of seconds and even minutes [4]–[6], a phenomenon that can be due to a combination of synaptic and intrinsic mechanisms. Following prolonged neuronal firing (seconds or tens of seconds), a long-lasting membrane hyperpolarization and a decrease in neuronal excitability has been attributed to the activation of a Na^+^-dependent K^+^ current [1], [7]–[11]. These long-lasting alterations of the membrane potential and conductance have then an impact on the response to subsequent incoming stimuli, such that a given stimulus will evoke a different response depending on the preceding events. Further, the activation of slow ionic currents has an effect that transcends the individual neurons to become a network effect. For example, if a sizeable proportion of the neuronal population is hyperpolarized due to a ionic current it translates in a decreased synaptic activity in the network and in a globally decreased excitability.

To understand how the intrinsic properties of the individual neurons modulate the cortical network operation, here we studied the influence of the membrane potential trajectory and resulting firing pattern during a stimulus response on the subsequent slow afterpotentials both *in vitro* and *in vivo*. We devised two protocols of stimulation (square *versus* sinusoidal current injection) as intracellular surrogates of continuous ON visual stimulus *versus* sinusoidal drifting gratings in a simple cell, or modulation by sinusoidal gratings in a complex *versus* a simple cell respectively. We find that the trajectory of the membrane potential during the stimulus and the temporal distribution of action potentials modulate the activation strength of slow afterhyperpolarization and afterdepolarization currents that determine the value of the membrane potential during several seconds after the stimulus.

## Materials and Methods

This research was approved by the CEIE (Comisión de Ética en la Investigación Experimental) Universidad Miguel Hernandez de Elche (Spain).

### Experiments *in vitro*

Cortical slices were obtained from 2- to 6-month-old ferrets of either sex that were deeply anesthetized with sodium pentobarbital (40 mg/kg) and decapitated. Their brains were quickly removed and placed in an ice cold solution (see below). Four hundred-micrometer-thick coronal slices of the primary visual cortex were cut on a vibratome [5]. A modification of the technique developed by [12] was used to increase tissue viability. After preparation, slices were placed in an interface-style recording chamber (Fine Sciences Tools, Foster City, CA) and bathed in ACSF containing (in mM): NaCl, 124; KCl, 2.5; MgSO_4_, 2; NaHPO_4_, 1.25; CaCl_2_, 2; NaHCO_3_, 26; and dextrose, 10, and was aerated with 95% O_2_, 5% CO_2_ to a final pH of 7.4. Bath temperature was maintained at 34–35°C. In experiments that required reduction of [Na^+^]_0_, all or part of NaCl was replaced with Choline-Cl; [NaHCO_3_] was not changed, giving a solution containing at least 26 mM Na^+^. The muscarinic blocker scopolamine (10µM) was added to prevent cholinergic effects of Choline-Cl. When calcium was reduced from 2.5 to 0.1 mM, Mg^2+^ was increased from 2.5 to 3.9 mM. Nifedipine was obtained from Sigma and applied locally via pressure ejection from a micropipette (1–4 µm tip diameter) after being dissolved in the bathing solution (500 µL). After the micropipette was positioned in proximity to a target cell in the slice, a brief pulse of pressure (10–250 msec; 250–350 kPa) was applied to the back of the microelectrode to extrude 5–20 pl of solution per pulse.

Intracellular recordings were initiated after 2 hr of recovery. Sharp intracellular recording electrodes were formed on a Sutter Instruments (Novato, CA) P-97 micropipette puller from medium-walled glass and beveled on a Sutter Instruments beveller to final resistances of 50–100 MΩ. Micropipettes were filled with 2 M potassium acetate. Recordings were digitized, acquired and analyzed using a data acquisition interface and software from Cambridge Electronic Design (Cambridge, UK). Resistive and capacitative artifacts were carefully compensated by bridge balance and capacity compensation respectively (Axoclamp 2B, Axon Instruments).

### Experiments *in vivo*

Intracellular recordings *in vivo* were obtained from the primary visual cortex of cats following the methodology previously described [13]. In short, adult cats were anesthetized with ketamine (12–15 mg/kg, i.m.) and xylazine (1 mg/kg, i.m.). The cat was then mounted in a stereotaxic frame and ventilated. A craniotomy (3–4 mm wide) was made overlying the representation of the *area centralis* in area 17. During recordings, anesthesia was maintained with propofol (5 mg/kg/h) and sulfentanil (8 µg/kg/h). The heart rate, expiratory CO_2_ concentration, rectal temperature, and blood O_2_ concentration were monitored throughout the experiment and maintained at 150–180 bpm, 3–4%, 37–38°C, and >95%, respectively. The EEG and the absence of reaction to noxious stimuli were regularly checked to insure an adequate depth of anesthesia. To minimize pulsation arising from the heartbeat and respiration, a cisternal drainage and a bilateral pneumothorax were performed, and the animal was suspended by the rib cage to the stereotaxic frame. After the recording session, the animal was given a lethal injection of sodium pentobarbital. All experimental animals were cared for and treated in accordance with the Spanish regulatory laws (BOE 256; 25-10-1990) which comply with the EU guidelines on protection of vertebrates used for experimentation (Strasbourg 3/18/1986). Intracellular recordings *in vivo* were obtained with identical micropipettes to the ones used to record from the cortical slices. Data recording and acquisition were made using the same methods as for the *in vitro* data.

### Electrophysiological analysis

In order to characterize the electrophysiological type of neurons we used 300–500 ms depolarizing current pulses. Longer periods of firing were induced by sinusoidal current injection at a frequency of 2 Hz for 20 sec or by depolarizing square pulses of 20 sec, with an amplitude 0.7±0.3 nA for depolarizing pulses and 1.0±0.5 nA for sinusoidal injection. The instantaneous frequency was computed as the inverse of the interspike interval. Responses to several repetitions of the current injection were averaged and they were quantified as spikes per cycle for sinusoidal current injections or in spikes per second for the square pulse current injections (500 ms bins). The slow spike frequency adaptation was measured as the firing rate decay between the first and the last 500 ms of the 20 sec of stimulation. Spike frequency adaptation during the pulse is referred to as “adaptation index”: the percentage of spikes during the last 500 ms of the protocol with respect to the number of spikes during the first 500 ms of either the pulse or the sinusoid; therefore the higher the adaptation index, the lower the adaptation. The decay time constant was determined by fitting an exponential to the 40 values corresponding to the average number of spikes per cycle (sinusoids) or to the mean frequency for 500 ms bins (square pulse). The amplitude of the afterpotential was taken at the peak of the AHP (minimal membrane potential value), which occurred between 35–45 ms after the end of the injection protocol. The duration was taken from the start of the AHP to the time when the membrane potential returned to the resting level. Given that these were very slow changes, those cases where the membrane potential did not return completely to the baseline were excluded of the duration measurement (n = 41). Data are reported as mean ± SD.

## Results

Intracellular recordings from a total of 116 neurons in visual cortex (99 *in vitro*; 17 *in vivo*) were included. Recordings were obtained from layers 2/3 and 4 in the cortical slices and from any layer while *in vivo*. Only neurons with overshooting action potentials and classified as regular spiking according to criteria in [14], [15] were included in the study. The reason for this selection was to have an homogeneous electrophysiological population and to be able to compare spike frequency adaptation across neurons.

**Figure 1:**
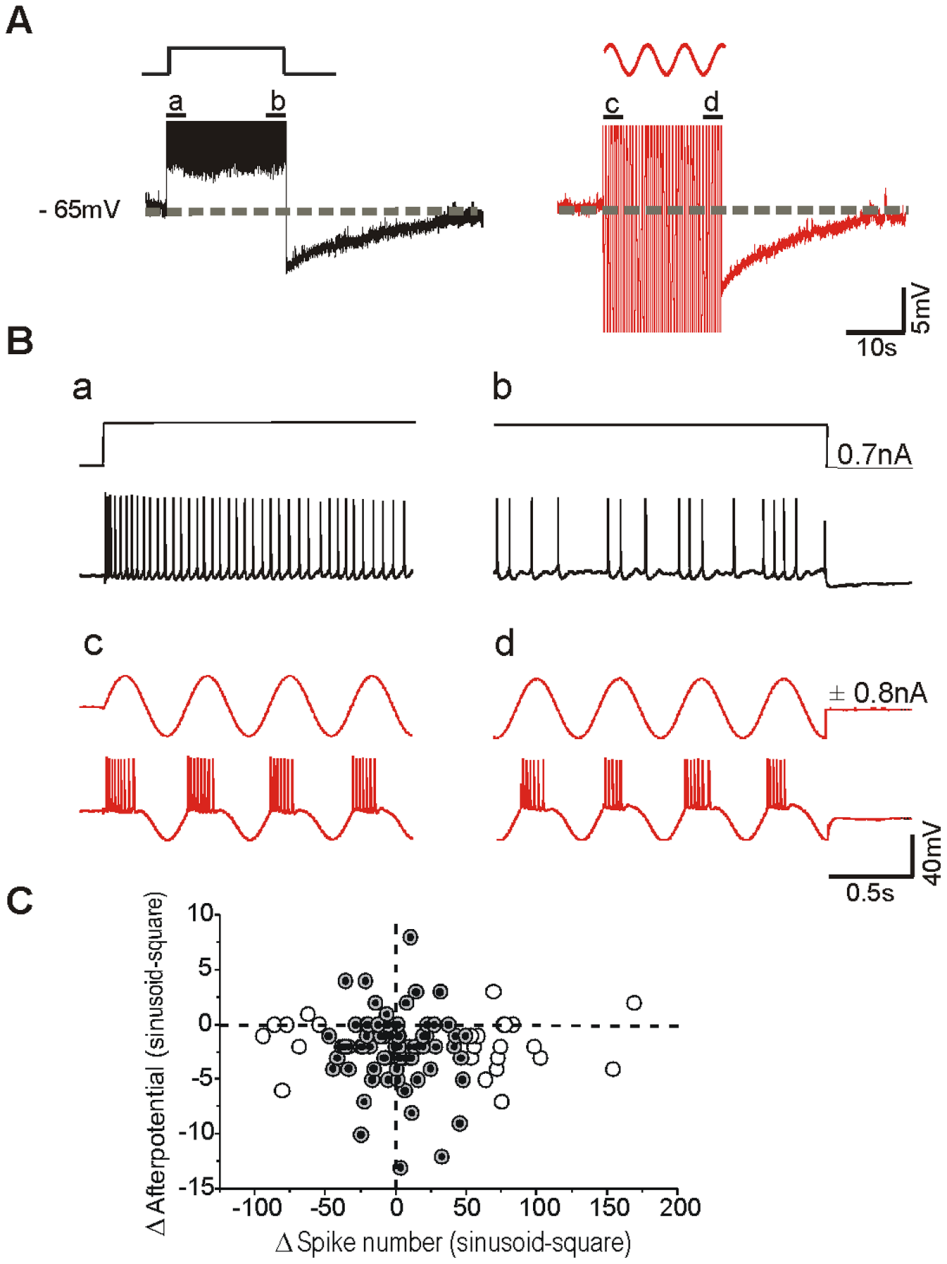
Slow postpotential induced by two different patterns of stimulation in visual cortex neurons *in vitro*. **A**. The postpotential evoked by the injection of a depolarizing square pulse of 20 sec of duration is compared against the one induced by injection sinusoidal current for 20 sec (right) in the same neuron recorded *in vitro*. The intensity (nA) of both stimuli was adjusted to evoke similar number of action potentials (245 spikes with the square pulse and 282 spikes with the sinusoid). In both cases, a slow afterhyperpolarization following the current injection was observed. A larger AHP (11 mV, 41 s duration) followed the injection of sinusoidal current than the square pulse (8 mV, 39 s duration). **B**. Expanded traces from A. **a, b, c, d**: Comparison of spike-frequency adaptation generated in response to intracellular injection of square pulse (**a, b**) and sinusoidal current (**c, d**). Notice that action potential discharge had adapted strongly at the end of the square pulse. **C**. Plot of the difference in the number of action potential evoked with both protocols for each of the neurons against the difference in afterpotential amplitude. Black point show the neurons with the difference of AP was ≤50.

Long depolarizations (of the order of tens of seconds) and the subsequently evoked trains of action potentials induced slow changes in the membrane potential, usually in the form of a long lasting hyperpolarization (AHP) as previously described in neocortical neurons [6], [11]. In order to study the influence of membrane potential trajectory during prolonged stimulation on the subsequent membrane afterpotential, we compared the response evoked by two different protocols: the injection during 20 sec of a square pulse of depolarizing current with that of sinusoidal current at 2 Hz (Fig. 1A). A square pulse evoked a train of action potentials with frequency adaptation that would vary between neurons (Fig. 1B,ab). A sinusoidal injection evoked repetitive series of action potential trains that also showed a variable decay in the firing frequency (Fig. 1B,cd). In order to compare the spike frequency adaptation and the afterpotential evoked by both patterns of discharge, the intensity of the current injection was adjusted such that the total number of evoked action potentials was similar with both protocols (ranging between 38 and 650 in different cells). The membrane potential was set before the stimulus at −65±5 mV by means of current injection, such that different traces would be comparable across neurons.

Figure 1 illustrates the recordings obtained from a cortical neuron *in vitro* that fired 245 spikes in response to the square pulse and 282 with the sinusoidal stimulus. Both protocols induced a certain degree of spike frequency adaptation during the discharge (Fig. 1B,ab for square pulse; 1B,cd for sinusoidal current) and were followed by a long-lasting slow hyperpolarization of the membrane potential (Fig. 1A). This neuron represents the most commonly observed case (see below): the afterhyperpolarization (AHP) evoked by the sinusoidal current injection was of larger amplitude (11 mV) than that evoked by the square pulse (8 mV). The duration of AHP was 41 s (sinusoidal current) and 39 s (square pulse).

Given the length of the protocols (20 s) and the different time course and intensity of the adaptation with both protocols, it was often difficult to adjust the intensity in order to evoke an identical number of action potentials. To rule out that a difference in the number of action potentials evoked by both protocols would determine distinct slow afterpotentials, we explored the relationship between these characteristics. Figure 1C illustrates the difference in the number of action potentials evoked with both protocols against the difference in afterpotential amplitude for each of the 99 neurons recorded *in vitro*. To the right of 0 on the x axis are the neurons in which the sinusoid evoked a larger number of action potentials (n = 57), and to the left of 0 those in which the square pulse evoked a larger number (n = 40), while in two cases there was no difference. There was no significant difference between the averaged differential afterpotential evoked in both groups respectively (−1.99±3.09; −2.08±3.30; p = 0.85) and no significant correlation was found between difference in action potential number and difference in afterpotential (R = −0.04).

### Spike-frequency adaptation during trains of action potentials

**Figure 2:**
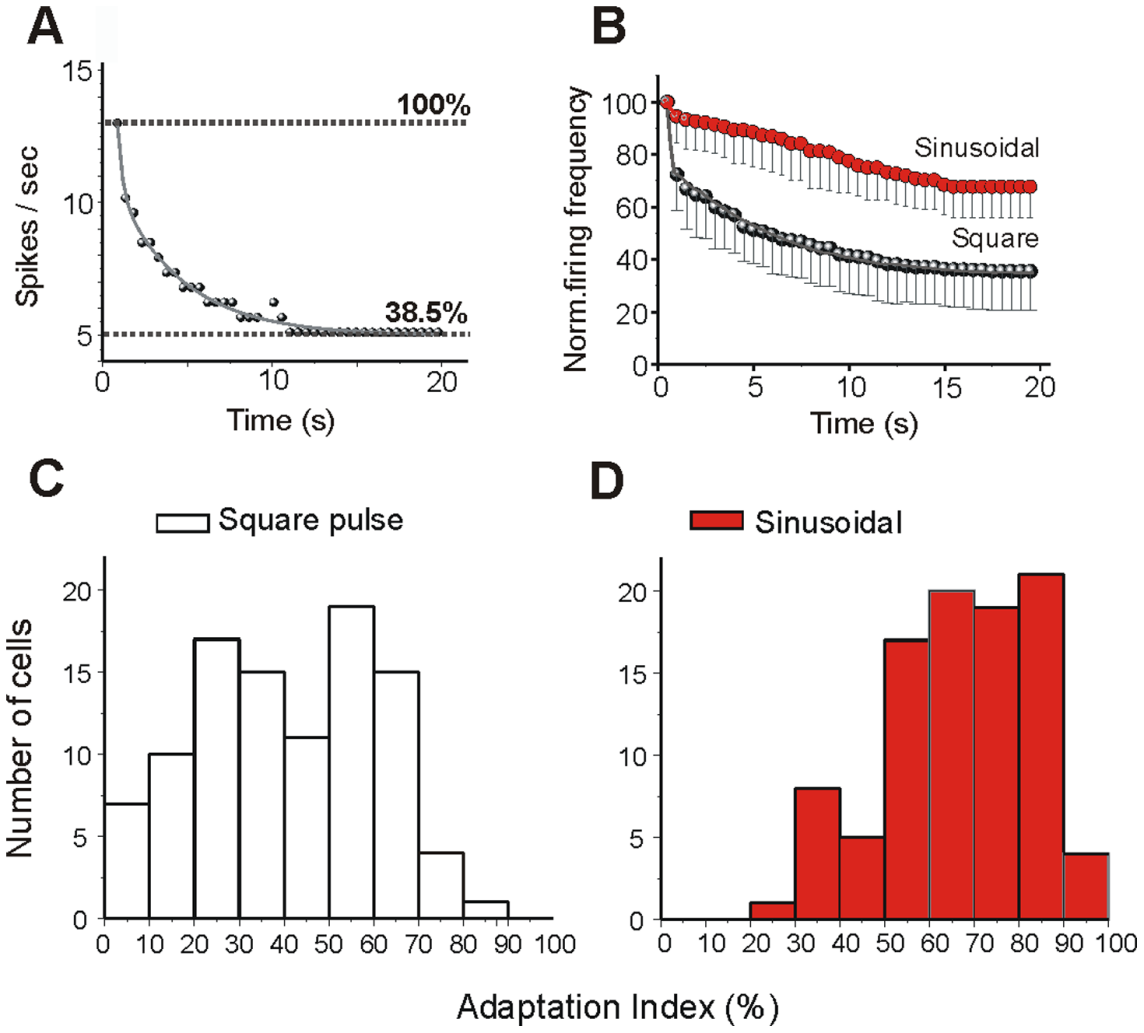
Slow adaptation during action potential discharge. The time course and strength of the firing rate adaptation varies with the type of intracellular injection current (square pulse or sinusoidal current). **A**. Example of the plot of the rate of the decay (fit by a single exponential) during square pulse intracellular injection current. The firing rate has been normalized such that 100% corresponds to the firing rate at the beginning of each stimulus. **B**. Comparison of the time courses and amplitudes of adaptation during the injection of each stimulus in 10 cortical neurons. Note a larger adaptation evoked by square pulse than sinusoidal injection current. **C, D**. Distributions of adaptation index values calculated for 99 cortical neurons recorded *in vitro* for square pulses (40±20%, C) and for sinusoidal current (67±18%, D). Adaptation index is the percentage of spikes during the last 500 ms of the protocol with respect to the number of spikes during the first 500 ms.

Spike-frequency adaptation was quantified for both protocols as described in Methods. Figure 2A shows how the discharge evoked by a square pulse in a neuron recorded *in vitro* adapted during the stimulus to 38.5% of the frequency evoked during the first 0.5 s. The distributions of the adaptation indexes measured in response to a square pulse and to a sinusoid in the 99 neurons recorded *in vitro* are shown in Fig. 2C and 2D. Square pulses produced a larger spike frequency adaptation (mean: 40±20%) than sinusoidal currents (67±18%; p<0.01 paired t test). Note that the firing frequency is averaged for the first 500 ms, and therefore fast adaptation that occurs during the first 500 ms is not taken into consideration.

In 10 neurons where *circa* 250 action potentials were evoked with each protocol, the firing decay was fitted to a single or a double exponential as shown in Fig. 2B. The time course of spike frequency adaptation was faster for square pulses than for sinusoidal current injection (Fig. 2B). The average decay in firing frequency evoked during square pulses had a fast component of 0.3±0.2 s time constant and a slower component of 10.2±5.1 s time constant. The time course of the firing frequency decay induced by a sinusoidal current injection could be fitted by a single exponential with an average time constant of 10.5±6.1 s. The slow components of both, the firing adaptation evoked by the square pulse and the sinusoidal current injection, were of the same order of magnitude (p = 0.92; *paired t test; p<0.001, n = 10*), and we speculate that they might both be mediated by the same ionic mechanism (see below).

### Membrane afterpotential following long action potential discharges

**Figure 3:**
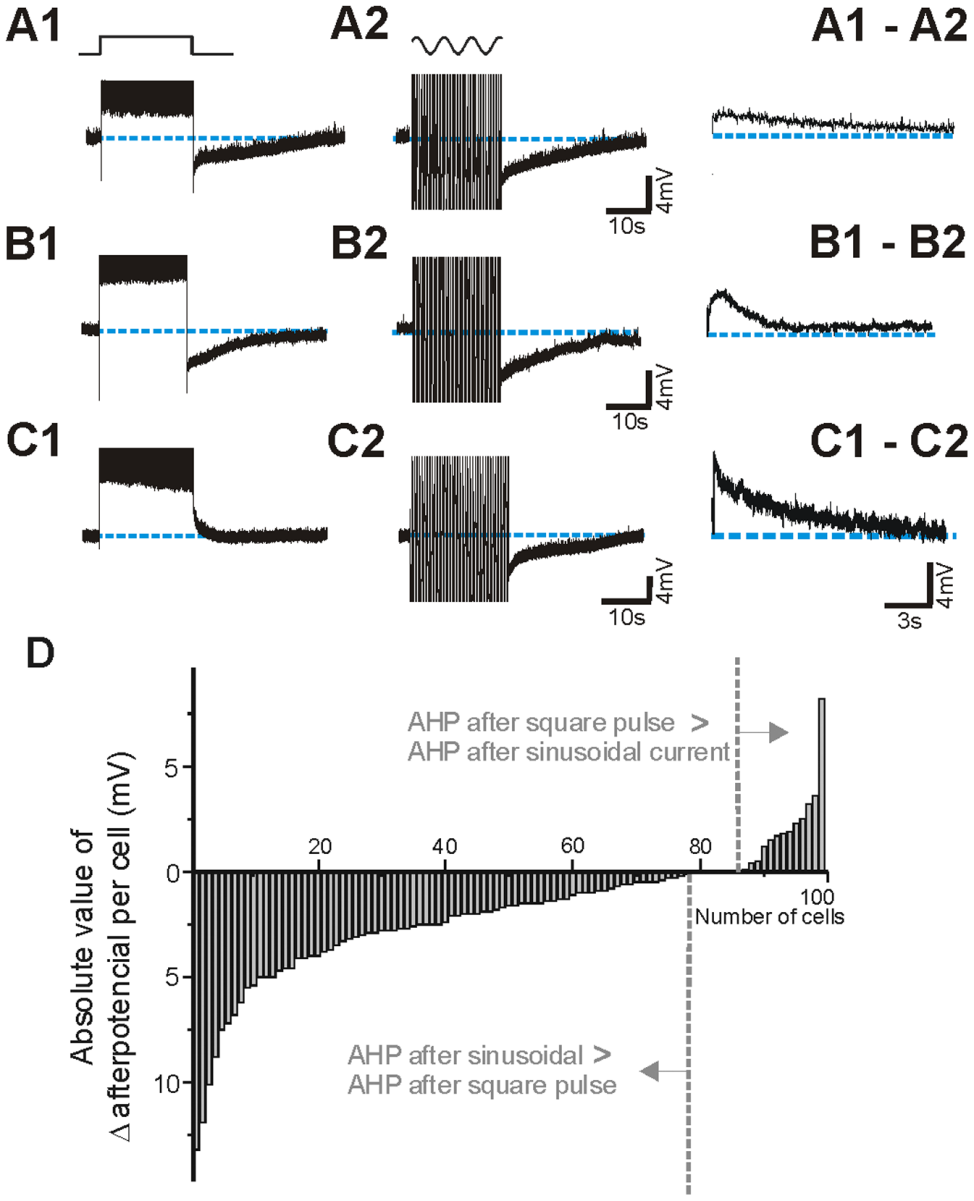
Postpotentials evoked by intracellular injection of depolarizing square pulses and sinusoidal currents. **A**, **B**. In the majority of the neurons recorded *in vitro* (99) we observed an AHP postpotential after the two protocols . **C**. In 6 neurons we observed an ADP after the square pulse stimulus and an AHP after the sinusoidal current. The left panels in A,B,C show the subtraction of both postpotentials (**1–2**). **D**. Absolute values of the postpotential differences for the 99 cortical neurons recorded *in vitro*. In 78 cases sinusoidal current generated a larger AHP, in 8 the postpotentials were similar and in 13 the AHP was larger after the square pulse.

As pointed out above, sinusoidal currents evoked larger AHPs than square pulses, a difference that was not correlated with the differences in the number of action potentials that both protocols evoked (Fig. 1C). Furthermore, in the 99 neurons studied *in vitro*, the average number of spikes triggered by both stimuli were not different (272±121 spikes for sinusoidal current and 263±127 spikes for square pulse; *p = 0.060*, paired *t-test*). Figure 3 illustrates different exemplar neurons representing the observed cases: a larger AHP following the sinusoidal injection (Fig. 3A), a similar AHP evoked by both protocols (Fig. 3B), and a depolarizing after-potential evoked by the square pulse and an AHP after the sinusoidal current injection (Fig. 3C).

To determine how these different behaviours were distributed across the neuronal population, a subtraction of the value of the AHP following the square pulse *minus* the one following the sinusoidal current injection (right hand panels in Fig. 3A–C) was calculated for the 99 neurons recorded *in vitro* and the values are presented in Fig. 3D. A larger AHP after sinusoidal stimulation was observed in 78 neurons; in 6 out of them a depolarizing afterpotential (ADP) followed the square current injection (Fig. 3C). In 8 cases both protocols evoked AHPs of similar amplitude and in 13 cases the square pulse evoked a larger AHP. The average amplitudes of the AHP were 8.5±3.3 mV for the sinusoidal current and 6.4±3.7 mV for the square pulse (p<0.001, *paired t-test*, n = 99).

In an earlier phase of the study, two more protocols of current injection were explored consisting of: 1) only the positive areas of the sinusoid (semicircles) and 2) 250 ms square pulses, both of them at 2 Hz like the sinusoidal frequency. Differently from the sinusoidal injection described in this study (Fig. 1Bc), no hyperpolarizing component was included in these protocols. Care was taken also to evoke similar number of action potentials with all four protocols. Our measurement of afterpotential revealed a value in between sinusoidal and the long square pulse. This information is provided in Figure S1 and suggests that the hyperpolarizing phase of the sinusoid is relevant to induce a larger negative afterpotential.

The measurement of the afterpotential duration was not always possible. Given that it is an event that lasts several seconds, the membrane potential does not always return right to the prepulse potential. In a representative sample (n = 58) in which resting membrane potential recovered completely, the duration of the AHP following the sinusoid was longer (33±17 s) than that following the square pulse (28±17 s; p<0.001, *paired t-test*).

### Intrinsic mechanisms underlying differences in slow membrane after-potentials

**Figure 4:**
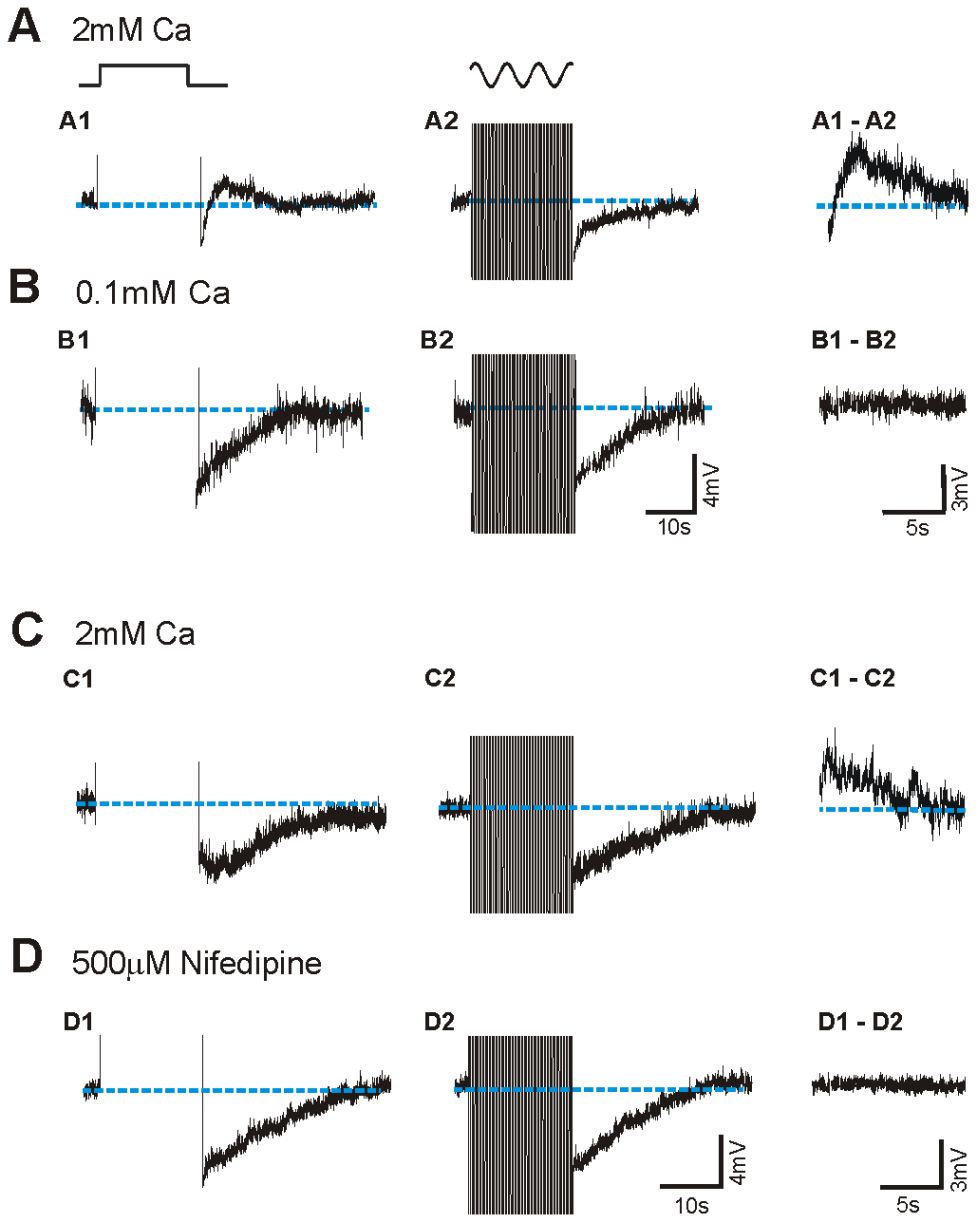
The difference in afterpotentials generated by pulses and sinusoids is calcium dependent. **A**. Intracellular injection of square pulse results in the generation of an ADP (**A**) and of sinusoidal current in a slow AHP (**B**). In low calcium (0.1 mM; B) the ADP disappeared and both protocols evoked similar AHPs (**A, B**). Left panels show the substraction of the afterpotentials. **C–D**. In a different neuron, local application of 500µM Nifedipine also abolished the differences in afterpotentials.

To explore the possible mechanisms that could contribute to a different afterpotential following long discharges, we selected neurons presenting large differences between the afterpotential following a square pulse and a sinusoidal current injection. We observed that after washing in low (0.1 mM) or nominal zero calcium solution the differences between both afterpotentials disappeared (n = 5). Fig. 4A–B illustrates a neuron that generated a small ADP after the pulse and removing calcium resulted in similar AHPs after both stimuli (Fig. 4B), similarly to the effect shown in [5; see Fig. 8].

In 6 cells the local application of the L-type calcium channel blocker nifedipine (500µm) also eliminated the differences in afterpotential evoked by both protocols (Fig. 4C–D). The average AHP amplitudes following both protocols while in nifedipine were not different (10±5 mV for square pulses; 9±4 mV for sinusoids; p = 0.127, *paired t-test*) whereas it was significantly different while in control solution (6 ± 2 mV for squared pulses; 9±3 mV for sinusoids; p = 0.016, *paired t test*). These findings suggest that the difference between the afterpotentials following both protocols is calcium-dependent and that the calcium entry during both stimuli may also be different.

The spike frequency adaptation could not be estimated under low calcium because the regular spiking firing was often transformed into some type of bursty firing [16] and the resulting adaptation was not comparable.

The sodium entry that accompanies action potential firing in neocortical neurons activates Na^+^-dependent K^+^ currents that generate a slow AHP [5], [7]–[10], [17] , which by its time course [18] coincides with the AHPs described here. We thus tested in 6 cells the effect of lowering [Na^+^]_o_ in the presence of 2 mM [Ca^2+^]_o_ (not shown). These cells showed average AHP amplitudes following both protocols which were 7.2±3.2 mV (for pulses) and 6.6±1.8 mV (for sinusoids); p = 0.504, *paired t-test*). In agreement with previous results [5] low [Na^+^]_o_ solution blocked the slow AHP, which was replaced by a slow ADP (+2.7±0.7 mV, square pulse; +1.9±1.3 mV, sinusoidal current; p = 0.054, *paired t test*).

### Intracellular recordings from visual cortex cells *in vivo*

Intracellular recordings were obtained *in vivo* from 17 neurons in cat visual cortex (area 17) classified as regular spiking according to criteria in [14], [15]. Similarly to that observed *in vitro*, spike frequency adaptation took place during firing with both protocols (square pulse and sinusoidal current) and was followed by a long-lasting afterpotential. Spike frequency adaptation was larger with square pulses (42±22%; n = 14) than with sinusoidal currents (69±15%; n = 7). Significant differences were found in 4 neurons in which we injected both protocols (44±20% and 64±19%, square and sinusoidal respectively; *p = 0.007, paired t-test*). In those 4 neurons, the number of action potentials evoked by both protocols were comparable (387 vs 406; 236 vs 277; 577 vs 685; 219 vs 183 spikes; square and sinusoidal respectively). Notice that there were 20 s current injections, and even though the intensity was regulated, evoking the same number of action potentials is virtually impossible unless one uses some close loop system.

**Figure 5:**
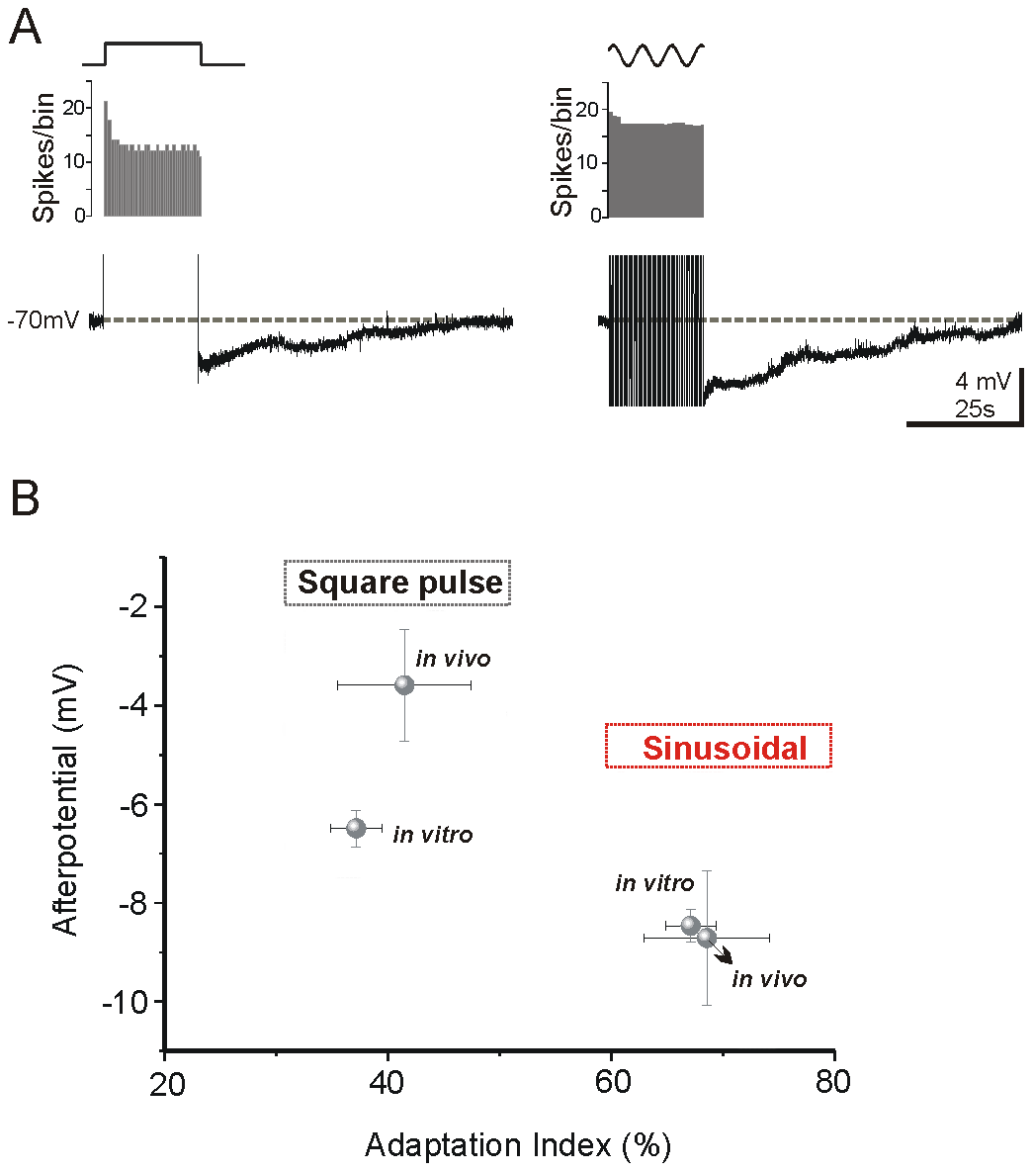
Comparison of firing adaptation and afterpotential measured *in vivo* or *in vitro*. **A.** AHPs recorded from a visual neuron *in vivo*: the square pulse evoked a smaller postpotential than sinusoidal current injection (4 mV and 49 s vs 6 mV and 54 s). **B**. Comparison between *in vivo* and *in vitro* conditions. Data are reported as mean ± SE. For sinusoidal current injection the values of adaptation index and postpotential was very similar in both conditions. *In vitro* conditions we observed a similar adaptation index from the same protocol but the postpotential was larger than *in vivo* condition.

*In vivo*, all 17 neurons showed some degree of afterhyperpolarization following each stimulus protocol, in contrast with the *in vitro* situation where 6 neurons showed an ADP after the square pulse. Similarly to what we observed *in vitro*, sinusoidal stimuli evoked a larger AHP (8.7±1.4 mV, n = 7), than square pulses (1.9±2.4 mV, n = 14), the difference between protocols being more prominent than *in vitro*. The duration of the slow AHP was longer than 30 seconds with both protocols, but the membrane potential fluctuations due to synaptic bombardment impeded its exact measurement. Figure 5A shows an example of a cortical cell recorded *in vivo* in which both protocols were injected in the same neuron. Sinusoidal current evoked an adaptation to 90% followed by an AHP with peak amplitude of 6.1 mV, whereas the square pulse resulted in a larger adaptation (74%) and smaller AHP (3.9 mV). It should be taken into account though that the number of action potentials evoked *in vivo* was in average much larger than *in vitro*, in particular 563±369 spikes evoked by the square pulse and 387±188 spikes evoked by the sine waves.

In summary, sinusoidal currents produced larger posthyperpolarizations and smaller spike frequency adaptations than square pulses both *in vivo and in vitro*. As a summary of this study, in Fig. 5B we show the relation of the average values of AHP amplitude (mV) and adaptation index (%) for the two protocols *in vitro* and *in vivo* conditions. When data from *in vivo* and *in vitro* experiments are compared, it can be seen that the degree of adaptation produced by either stimulus was similar in both conditions, but whereas AHPs evoked by sinusoidal currents were also similar, those produced by square pulses were larger *in vitro* than *in vivo* (Fig. 5B).

## Discussion

We draw two main conclusions from the data presented here. First, not only the spike firing but also the trajectory of the membrane potential during stimulation is critical to determine the properties of the afterpotential (amplitude and duration). This afterpotential in turn modulates the excitability of neurons for tens of seconds or even for minutes, affecting the subsequent pattern of discharge. Second, a calcium dependent current that is activated more efficiently by *plateau* than by intermittent depolarizations causes profound differences in the afterpotentials evoked by the two different stimulus patterns. Similar intrinsic mechanisms to the ones that we describe here would be at play when these membrane trajectories are achieved by means of visual stimulation. For example sinuoidal drifting gratings induce a sinusoidal modulation of the membrane potential in simple cells and a continuous depolarization in complex cells. A critical difference though is that in the current study we have explored the effects at the single neuron level, in the context of a silent network and thus in the absence of synaptic activity in order to identify the contribution of ionic mechanisms.

The two protocols used in this study allowed us to identify to what extent the time course and amplitude of afterpotentials are due to the trajectory of membrane potential during stimulation or to the total number of action potentials (see Fig. 1).

### Spike frequency adaptation and afterpotentials in square versus sinusoidal injection

We evaluated the adaptation and postpotential in each protocol for a similar number of action potentials generated during the stimulation and found significant differences in the spike frequency adaptation and the afterpotential between both protocols (Fig. 2). A large spike frequency adaptation was evoked with a *plateau* stimulation in the form of a square pulse, the decay of firing frequency was faster and the adaptation index was significantly higher than for the sinusoidal stimulation. On the other hand, the amplitude and duration of the afterhyperpolarization was larger with sinusoidal modulation of the membrane potential, as well as when we removed [Ca^2+^]_o_ or blocked L-type calcium channel both protocols evoked equal AHP.

It is somehow paradoxical that the protocol that induced a greater and faster spike frequency adaptation (the square pulse) is that followed by a smaller AHP, while the opposite occurs with the sinusoidal stimuli (less spike frequency adaptation and more AHP). This paradox may have a simple explanation. The spike frequency adaptation that occurs with the square pulse has a fast component absent in the sinusoidal firing. This can be seen in Fig. 2B (*in vitro*) and Fig. 5A (*in vivo*). The slow time course of the spike frequency adaptation is similar with the two protocols (Fig. 2B). As a result of the pattern of spike frequency adaptation during the square pulse, the actual firing rate during the second half of the protocol is lower with this protocol than with the sinusoids (Fig. 1B). Lower firing rates translates into lower sodium accumulation, thus activating the sodium- dependent potassium current less efficiently and giving it time to recover from earlier accumulation (for a model of this process see Fig.1C in [18]).

On the other hand, there is activation of the Ca^2+^-dependent ADP that follows the *plateau* depolarization, thus reducing the amplitude of the AHP [5]. This slow ADP was observed occasionally in control situation and revealed in low Na^+^ (Fig. 1, 3). It was extinguished by reduced transmembrane Ca^2+^ currents as a result of decrease [Ca^2+^]_o_ and also by block of L-type calcium channels. Decrease or elimination of the ADP resulted into an increase of duration and lengthening of the slow AHP (Fig. 4; see Fig. 7 and 8 in [5]) [19]–[21]. The fact that the ADP is larger following the square pulse and it is sensitive to L-type calcium channel blockers suggests that calcium is entering neurons through L-type channels in larger amounts during long than during intermitent depolarizations. Calcium can enter through L-type calcium channels not only during spikes [22] but also during the depolarization of the long pulses, given that the membrane potential values reached (around −40 to −35 mV) is enough to open these calcium channels [22], [23]. In conclusion, both slow components, AHP and ADP are modulating the trajectory of the membrane potential of the neuron for tens of seconds or even for minutes (Fig. 3), therefore affecting the subsequent pattern of discharge and the interactions with the rest of the neurons of the cortical network.

According to these experiments, the scenario that we propose is the following: the repetitive firing in both cases induces a total sodium entry with action potentials that could be similar given the similar number of action potentials, although its dynamics would vary with the distribution of firing over time. The prolonged activation of cortical neurons is followed by a slow AHP in the majority of neurons (Fig. 1, 3). Several investigations have demonstrated prolonged afterhyperpolarizations after repetitive action potential generation often mediated by Na ^+^ or Ca^2+^ -activated K^+^ currents [7]–[11], [17], [18], [24]. Somatic current injections elicit action potentials that induce Ca^2+^ transients in the soma and proximal dendrites [25], [26]. Ca^2+^ entry during repetitive firing serves as a feedback regulator of firing rate [27], activating Ca^2+^ -dependent K^+^ channels that produce spike frequency adaptation. This calcium would also activate the calcium-dependent cationic current that counteracts the AHP: a large entrance of Ca^2+^ would facilitate the activation of Ca^2+^ sensitive depolarization currents –such as a cationic conductance. The Ca^2+^-sensitive cationic current is not activated when [Ca]_o_ is lowered or it is blocked by the L-channel calcium antagonist nifedipine in agreement with our present results (Fig. 4). The values of membrane potential reached during depolarization –not only during action potential firing- are well within the wide range of activation of the L-type channels. However, the depolarized *plateau* membrane potential caused by the long depolarizing pulses would allow a larger calcium input than sinusoidal pulses, given that with sinusoids there are hyperpolarizing phases half the time.

Sodium enters neurons with action potentials and activates in both protocols Na^+^ -dependent K^+^ currents that generate a slow AHP [7]–[11], [17], its time course coincides with the described in our data. In agreement with previous results [11] the effects of lowering [Na^+^]_0_ in the presence of 2 mM [Ca^2+^]_0_ also resulted in a block of the slow AHP, and in our case in both protocols the slow AHP was replaced by a slow ADP (n = 6). The average postpotential was 2.9±0.8 (square pulse) versus 1.8±1.2 mV (sinusoidal current) and no significant differences were found between both protocols in lowering [Na^+^]_0_.

While work in slices allows procedures that are not feasible *in vivo*, such as ionic substitution and exposure to channel blockers in controlled conditions (e.g. Fig. 4), studies *in vivo*, albeit more descriptive in nature, permit the characterization under natural conditions of intense background synaptic activity and some degree of neuromodulation (Fig. 5A). With the aim of exploring the validity of our observations *in situ*, we reproduced the two stimulation protocols in intracellularly recorded neurons in V1 *in vivo*. For the square pulse, the adaptation and slow postpotential observed *in vitro* was very similar to *in vivo* condition. In the case of sinusoidal current injection the amplitude of the slow AHP was smaller while *in vivo* (Fig. 5). This difference may be explained by differences in the cell types examined (*in vitro* was restricted to superficial layers), the lack of spontaneous activity *in vitro*, or differences in the presence of modulatory neurotransmitters [7].

### Differences between Regular Spiking (RS) and Fast Spiking (FS) neurons

We described in a previous paper [6] the existence of spike-frequency adaptation in FS neurons in response to 20-s-long square depolarizing pulses and sinusoidal currents (2 Hz), even though these neurons were classically defined as *non-adapting* neurons. FS neurons recorded *in vitro* showed slow frequency adaptation with a time constant in the range of seconds for both sinusoidal current injections (8.8±5.8 s) and square depolarizing pulses (6.7±3.3 s). When comparing across different neuronal electrophysiological types we concluded that the average firing decay time is significantly faster for RS neurons than for FS neurons. The reduction of the firing was similar in FS neurons in response to both protocols with an adaptation index of 50% after an stimulation of 20 s. In the present data (RS neurons) we found a larger adaptation index (39.5% for square pulse). Following long discharges, FS neurons recorded *in vitro* displayed a slow AHP smaller than RS neurons *in vitro* (duration ≤23 s; 5.5±2.7 mV). These differences are probably due to the existence of adaptation currents with faster time course such as calcium-dependent potassium currents in non-FS cells [28]–[31].

As described for RS neurons, the strength of adaptation in FS neurons was less *in vivo* (30% for the sinusoids, 40% for the pulse) than *in vitro* (44% for the sinusoids, 53% for the pulse). One explanation is the possibility that ongoing activity *in vivo* may have an effect on the steady state of adaptation [32]: a fraction of Na^+^ -dependent K^+^ current may be activated due to the presence of spontaneous activity *in vivo*, such that neurons are already partially adapted in the baseline condition. On the other hand, the lack of spontaneous activity in slices would result in neurons that have not been “pre-adapted.” This implies that FS and RS neurons *in vivo* possess the same slow conductance as they do *in vitro*, but the basal state of their activation may be different in the two instances. Thus a neuron's ability to be in different states of adaptation suggests a mechanism that may allow the neuron to integrate new inputs while still tracking its firing history. In addition, it is reasonable to suggest that adaptation *in vitro* may be more prominent than *in vivo* owing to a decrease in efficiency for meeting the metabolic demands of the neurons.

### Effects in the cortical network function

The slow adaptation in a population of inhibitory and excitatory neurons has important consequences for the computational properties of the cortical network. Recent studies demonstrate the homeostatic role of adaptation on visual processing [33], not only equalizing responses but also facilitating independence in selectivity across the population by decorrelation [18], [34]. Neurons *in vivo* slowly adapt when they are stimulated for extended periods of time, i.e., in response to sustained high contrast stimulation [4], [11], [33], [35]–[39] or as a result of long-lasting current injection [5], [6], [40]. Furthermore, the spontaneous activity displayed by primary sensory neurons (A1) in the awake animal, when mimicked *in vitro*, is enough to induce a significant adaptation by activating potassium channels (see Fig. 7 in [41]). The activation of ionic channels generating slow adaptation currents has an effect on the network that can be considered equivalent to synaptic depression or inhibition activated in recurrent circuits, such that the spike discharge is regulated by the neuron's own previous activity. In the context of the cortical network, intrinsic adaptation has also an interaction with synaptic plasticity phenomena resulting in additional computational capabilities. For instance, the interaction of adaptation and short term plasticity such as synaptic depression results in the computation of rate of change and of anticipation [42], [43]. Synaptic depression and spike frequency adaptation are both activity-dependent mechanisms and they can yield similar effects at the network level. For example, both mechanisms have been shown to be powerful mechanisms of gain control [40], [44]. Both excitatory and inhibitory neurons share similar mechanisms for slow adaptation, what should contribute to the excitatory/inhibitory balance within the cortical network [45]. The impact of long lasting adaptation (tens of seconds) on visual processing is not as well known as the one of brief adaptation. A recent study exploring this reveals that long adaptation (40 s) did not induce stronger effects than a short one (0.4 or 4 s), however the recovery was notable slower [36]. Similar time course of sensory adaptation can be observed as well in the awake animal [41]. Our study should contribute to the understanding of the ionic mechanisms contributing to this phenomena. Still, when the animal is awake and behaving, it is possible that what is a long lasting stimulation in the anesthetized animal, becomes closer to an intermittent stimulation thanks to the microsaccades [46], [47]. In that sense, the sinusoidal stimulation used in this study would be closer to the visual activation during an active exploration of the visual landscape.

## Supporting Information

Figure S1 **Value of afterpotential following different 20 s protocols.** **A**. Values of afterpotential (see Methods) for sinusoidal injection (n = 27), positive area of the sinusoid (half-moons) (n = 15) , square pulses (n = 25) –all of them at 2 Hz- and long square pulse (n = 17). Out of them, only the sinusoidal pattern included injection of hyperpolarizing current. In this case, not all neurons had all for protocols done. **B**. Same protocol as in A but for a selection of 15 neurons that had all 4 protocols done. In all cases, the intensity was adjusted such that a similar number of action potentials was evoked in each neuron. The average numbers of action potential for the 4 protocols displayed were 230, 211, 223 and 230 from left (sinusoid) to right (long square pulse).
(TIF)
