# Supplementary figures and images for: Adaptation in the Visual Cortex: Influence of Membrane Trajectory and Neuronal Firing Pattern on Slow Afterpotentials
Source: PLoS One. 2014 Nov 7;9(11):e111578. doi: 10.1371/journal.pone.0111578 (PMC4224415; doi:10.1371/journal.pone.0111578)

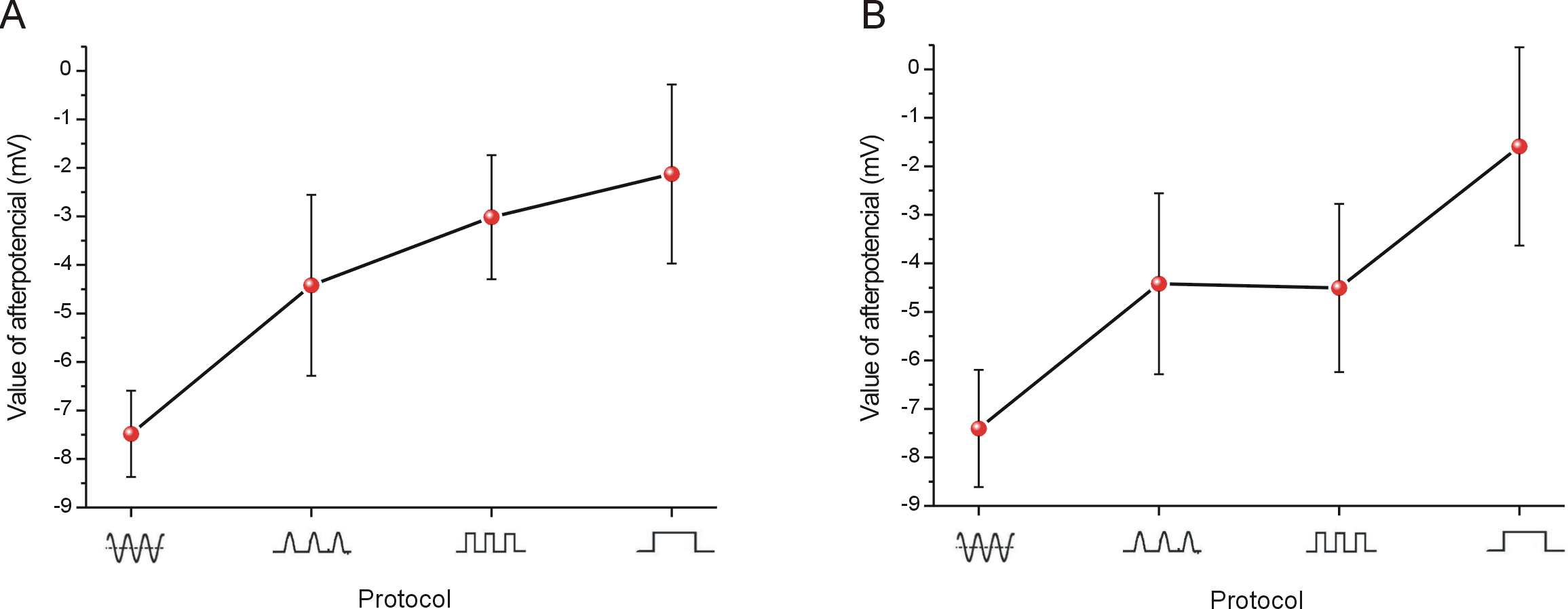

Supplement: Figure S1 — Value of afterpotential following different 20 s protocols. A. Values of afterpotential (see Methods) for sinusoidal injection (n = 27), positive area of the sinusoid (half-moons) (n = 15) , square pulses (n = 25) –all of them at 2 Hz- and long square pulse (n = 17). Out of them, only the sinusoidal pattern included injection of hyperpolarizing current. In this case, not all neurons had all for protocols done. B. Same protocol as in A but for a selection of 15 neurons that had all 4 protocols done. In all cases, the intensity was adjusted such that a similar number of action potentials was evoked in each neuron. The average numbers of action potential for the 4 protocols displayed were 230, 211, 223 and 230 from left (sinusoid) to right (long square pulse). (TIF) [file pone.0111578.s001.tif]
